# Platelet Donation by Apheresis for Female Blood Donors with Low Hemoglobin or Iron Deficiency

**DOI:** 10.1159/tmh/aevag005

**Published:** 2026-10-10

**Authors:** Laura Infanti, Vildana Pehlic, Thomas Volken, Gregor Stehle, Andreas Holbro, Andreas Buser

**Affiliations:** a Regional Blood Transfusion Center Northwestern Switzerland, Swiss Red Cross, Switzerland; b Department of Hematology, University Hospital of Basel, Basel, Switzerland; c University of Basel, Basel, Switzerland

**Keywords:** Blood donors, Plateletpheresis, Anemia, Iron deficiency

## Abstract

**Introduction:**

In Switzerland, platelet (PLT) donation is allowed with hemoglobin (Hb) ≥110 g/L and ≥120 g/L for women and men, respectively. We report on a policy of proposing PLT apheresis to female donors no longer eligible for whole blood (WB) donation because of low Hb and/or iron deficiency.

**Methods:**

We assessed 85 women with Hb <125 g/L and/or ferritin ≤ 15 µg/L who changed to PLT apheresis between 01/1997 and 12/2023 and underwent at least one PLT apheresis/year. Hb and ferritin were evaluated at the first WB donation, at the first and last PLT apheresis and were estimated as adjusted values over 30 PLT donations. Donor retention over a 24-month period was evaluated.

**Results:**

The switch to PLT apheresis occurred after a median of 3 (range: 1–33) WB donations. In 42 women (49%), both Hb and ferritin were low, 32 (38%) had low ferritin, and 11 (13%) low Hb only, respectively. Median age (range) at first WB donation and at last PLT donation was 30 years (18–53 years) and 45 years (19–68 years), respectively. Median (range) Hb and ferritin were 134 g/L (120–153 g/L) and 24 µg/L (2–177 µg/L) at the first WB donation and 130 g/L (103–154 g/L) and 14.5 µg/L (3–113 µg/L) at the last PLT apheresis. Over the course of 30 PLT donations and adjusted for donor age, an increasing trend was observed for both Hb (mean + 0.26 g/L; 95% CI: 0.16, 0.35; *p* < 0.001) and ferritin (mean + 0.48 µg/L; 95% CI: 0.21, 0.75; *p* < 0.001). At the end of a 24-month period, 94% (95% CI: 86.4%, 97.5%) women were still donating.

**Conclusion:**

Women with low Hb and/or iron deficiency benefit from a change to PLT donation and can be retained as regular, highly committed PLT donors.

## Introduction

Low hemoglobin (Hb) is a frequent reason for deferring blood donors and a major cause of losing active volunteers permanently. Iron deficiency (ID) represents both the principal cause of low Hb [1] and the most frequent and relevant undesired side effect of blood donation with a potentially negative impact on donor health. Avoiding substantial iron loss and preventing anemia in blood donors is a crucial issue not only for reducing deferrals and preserving the pool of active donors but also because protecting healthy volunteers represents an ethical responsibility of the blood collection centers [2–4].

Because Hb levels alone do not reflect iron stores adequately, many blood institutions monitor ferritin in donors according to local policies. In the context of blood donation, the ferritin limits adopted for defining ID are mostly those indicated by the WHO (≤15 µg/L) [5], while values of 25 µg/L to 30 µg/L are widely used for the diagnosis of ID in the clinical setting. Both the US and the European regulations indicate ferritin monitoring as one of possible measures for preventing anemia in regular donors. Expanding the interval between donations for individuals with low ferritin [6], providing iron supplementation, or combining both measures [7–10] are examples of ferritin-based policies that were proven to be effective in reducing ID in blood donors. The recently nationwide implemented strategy in the Netherlands showed that longer intervals between whole blood donations in persons having low ferritin but acceptable Hb levels are effective in decreasing the number of Hb deferrals but had an overall negative impact on donor return and on blood procurement [6]. A further possible approach is proposing platelet (PLT) or plasma apheresis instead of whole blood donation (that causes the loss of up to 250 mg iron) to donors already having or being at risk of developing low Hb and/or low ferritin.

PLT apheresis is one key activity of many blood donation centers. In Europe, while the number of transfused PLT units increased in the last years [11], blood establishments face a general shrinking and aging of the blood and PLT donor base. Ensuring an adequate PLT supply to hospitals can be challenging because PLT components can be stored for a maximum of 7 days and because requirements often show a wide short-term variability. Therefore, much effort is needed for both recruiting suitable volunteers for PLT apheresis and for retaining regular and reliable PLT donors in the long term. Because of the very low iron loss associated with single apheresis [12] and because PLT and plasma are highly required blood products, proposing apheresis donation is likely to benefit both the donors at risk of anemia and ID and the blood centers.

Blood donors at particularly elevated risk of ID and anemia are men and women aged 16–25 years, women in childbearing age and subjects with Hb levels close to the lower acceptable value [13]. With each PLT apheresis, iron is lost principally with the red blood cells remaining in the disposables and with the samples drawn for routine testing [12], and high-frequency PLT donation can also lead to low Hb and ID. A large US study showed that repetitive PLT apheresis was an independent risk factor for iron depletion in men who had donated exclusively PLT for the most recent 2-year period [14]. Similarly, ID and/or anemia were described in frequent PLT donors belonging to different ethnic populations [15–17]. Overall, however, if PLT apheresis is performed at the maximum frequency defined by regulations, the impact on iron stores appears to be limited [12, 18]. For this reason, some national blood services allow PLT apheresis also if ID is present or if Hb levels are inadequate for whole blood donation. Following this approach, data from the USA showed that lowering the minimum Hb to 115 g/L allowed to increase the number of available PLT donors without being associated with a Hb decline over time [19]. Similarly, in a Swiss retrospective study on 302 PLT donors with Hb ≤ 125 g/L, no significant Hb decrease was observed over a 2-year period of repetitive apheresis and with donation intervals of less than 60 days [20]. Neither study reported on ferritin assessments.

While setting the same Hb cutoff of 125 g/L for females and 135 g/L for males for all type of donations, the European regulations allow PLT donation with lower Hb values after consultation with the responsible physician or as established by a competent authority [21]. In line with this policy, the Swiss national Blood Service accepts Hb values of ≥110 g/L and ≥120 g/L for PLT apheresis in females and males, respectively, after medical evaluation [22].

At our center, a complete blood count on a venous blood sample and a ferritin measurement are performed in all first-time donors, every 2 years in regular whole blood donors and at least yearly in regular PLT donors. Subjects with low Hb and ferritin values <15 µg/L undergo a medical interview and an additional Hb or ferritin check according to medical judgment. Based on the results, donors can be deferred for 6–12 months, advised to consult the general practitioner for further investigations or iron supplementation or are asked to change to PLT apheresis.

As a first evaluation of this policy, we conducted a retrospective analysis of 43 subjects, both males and females, with Hb below the acceptance limits for whole blood donation and/or low ferritin who donated PLT between 1997 and 2008 at our center and demonstrated an increase of both Hb and ferritin over 11 years of regular PLT donations [23]. Hereby we present an update of the longitudinal analysis of this strategy focusing on the group of regular female PLT donors.

### Subjects and Methods

This is a single-center, retrospective analysis of data collected between January 1997 and December 2023 at the Regional Blood Transfusion Centre of Basel.

#### Study Cohort, PLT Donations, and Evaluated Data

The study cohort comprised women who were no longer eligible for whole blood donation having Hb values < 125 g/L or were advised against further donation because of low ferritin. All subjects were asked to change to PLT apheresis (no plasma apheresis is performed at our center). Included in the analysis were female donors who fulfilled all the donation eligibility criteria according to medical evaluation (i.e., not suffering of chronic medical conditions, relevant gynecological or other causes of blood loss, or ID/anemia) and with at least one PLT apheresis/year following the change of the donation type. Over the evaluation period, national regulations regarding both acceptable Hb levels and PLT donation frequency as well as our internal policy of ferritin measurement and donor counseling were unchanged. PLT aphereses were performed with the Amicus (Fresenius Kabi, software version 4.4 from January 2021) or the Trima (Terumo BCT, software version 7.0 until December 2023) according to standard operating procedures. As per internal policy, the interval between PLT aphereses was set at 6–8 weeks, with shorter intervals being possible depending on PLT shortages or specific requests (e.g., HLA matched PLT products). Within the cohort, three groups were defined based on the reason for the change to PLT donation: low Hb only (LowHb), both low Hb and ferritin (LowHbFerr), and low ferritin only (LowFerr).

Data analysis included donor age, Hb, and ferritin at the first whole blood donation, at the first PLT donation (switch), and at the last PLT apheresis; the total number of PLT apheresis per donor; the total number of PLT products obtained; the number and the reasons for temporary and definitive deferrals. Information on whether the study subjects took iron substitution was not consistently documented; therefore, these data were not evaluated.

#### Laboratory Measurements

As described above, a complete blood count (Sysmex 350 counter, Sysmex Digitana AG, Horgen, Switzerland) and a ferritin measurement (Architect ci8200 or Alinity I, Abbott Laboratories, IL, USA) were regularly performed in all donors. Ferritin was measured in PLT donors at individual intervals based on previous values and on medical evaluation. A cutoff ferritin of ≤15 µg/L was adopted for defining ID. Donors with ID were individuated on the low-ferritin list that was regularly generated from the IT database and viewed by a physician. Women and men with Hb ≤ 125 g/L and ≤ 135 g/L, respectively, and those found to have low ferritin underwent a medical interview and an additional Hb or ferritin check according to medical judgment.

#### Study Outcomes

The outcomes of the study included the differences in Hb and ferritin levels at the three distinct time points of first whole blood donation, switch to PLT donation and last PLT donation; the changes of Hb and ferritin during repetitive PLT apheresis; the overall retention of active PLT donors.

### Statistical Analysis

The characteristics of the study participants and the donation-related data were analyzed as median and range or mean and SD, as appropriate. We investigated differences in Hb and ferritin values at the first whole blood donation, at the switch, and at the last PLT apheresis with the Wilcoxon signed-rank test and two-sided sign test for paired samples. *p* values are reported also as Bonferroni-corrected *p* values, adjusting for multiple comparisons. Moreover, we conducted a sensitivity analysis within the Wilcoxon framework, contrasting the sample of donors aged 18–50 years over the entire donation period with the sample of those 50 years or older to assess the impact of pre- and post-menopausal status on the Hb and ferritin changes. We used generalized estimating equation models of the Gaussian family with robust standard errors, which adjust for repeated measures of the same subject, to evaluate Hb and ferritin levels over up to 30 PLT donations. Estimates of generalized estimating equation models including 95% confidence intervals (95% CI) are also reported adjusting for the reason of switching to PLT apheresis and for age (binary variable being 0 if age over the entire observation period was < 50 years and 1 otherwise). Finally, donor retention was evaluated as the proportion of active PLT using the non-parametric Kaplan-Meyer estimator. Donors were considered at risk when PLT apheresis was started and were censored either once they stopped donating PLT for any reason or at the end of the observation period. The whole single-failure-per-subject sample comprised 85 donors, representing 2,257 observations and a total of 8,600 months at risk. However, we limited the analysis of retainment to a period of 24 months to prevent censoring of donors with a shorter donation activity because they switched to PLT apheresis later during the observation period. Statistical significance was established at *p* < 0.05, with *p* < 0.10 being considered as borderline significant. Analyses were performed using the Stata package version 15.1 (StataCorp, College Station, TX, USA).

## Results

In reviewing retrospectively all PLT aphereses performed from January 1997 to December 2023 at our center, we identified 85 women who were counseled to change from whole blood to PLT donation. As shown in Table 1, the median number of whole blood donations before the switch was 3 (range: 1–33). The reason for changing the type of donation was Hb < 125 g/L and ferritin < 15 µg/L in most subjects (group LowHbFerr; 42 donors; 49%), followed by ferritin ≤ 15 µg/L with adequate Hb (group LowFerr) and by Hb < 125 g/L with ferritin > 15 µg/L (group LowHb). The median age of the cohort increased from the first whole blood donation (30 years; range: 18–53 years) to the last PLT donation (45 years; range: 19–68 years), and the proportion of donors younger than 50 years decreased accordingly from 96% at the first blood donation to 58% at the last PLT donation. Ferritin measurements were missing in 24 subjects (28%) at the first whole blood donation and in 25 (29%) at the last PLT apheresis, while 8 donors (9%) were not tested at their first PLT donation. Hb was below 110 g/L in three donors. One woman had Hb of 106 g/L at her first PLT donation, which was most probably an erroneous measurement, being her values 126 g/L three months before and 131 g/L 1 one month later. Two women had Hb of 109 g/L and 106 g/L at their last PLT apheresis and ferritin below 10 ng/mL at the same time, displaying ID anemia.

**Table 1.** Characteristics of the study cohort and changes of age, hemoglobin, and ferritin

Female blood donors in the cohort, *N* = 85			
Whole blood donations prior to switch, median (range), *n*		3 (1–33)	

Reason for switching to PLT donation			
Hb <125 g/L (group LowHb)	11 (13%)		
Hb <125 g/L and ferritin ≤ 15 ng/mL (group LowHbFerr)	42 (49%)		
Ferritin ≤15 ng/mL (group LowFerr)	32 (38%)		

	At the first whole blood donation	At the first PLT apheresis	At the last PLT apheresis
Age, median (range), years	30 (18–53)	33 (18–57)	45 (19–68)
Donors aged 18–50 years, *n*	82 (96%)	78 (92%)	49 (58%)
Hemoglobin, median (range), g/L	134 (120–153)	123 (106–149)***/***	130 (103–154)***/***
Aged 50+ years at last apheresis	133 (121–145)	124 (111–147)***/***	130 (117–151)***/***
Aged 18–50 years at last apheresis	136 (120–153)	123 (106^**§**^–149)***/***	130 (106^a^–154)**/*
Ferritin, (range), ng/mL	24 (2–177) missing: 24 (28%)	8 (2–23)***/*** missing: 8 (9%)	14.5 (3–113)***/*** missing: 25 (29%)
Aged 50+ years at last apheresis	18 (3–92)	7 (3–23)*/*	15 (7–113)***/***
Aged 18–50 years at last apheresis	24 (2–177)	8 (2–20)***/***	13 (3–42)**/**

All *p* values refer to Wilcoxon signed-rank test for first whole blood donation versus first PLT apheresis and first PLT apheresis versus last PLT apheresis. We reported unadjusted and Bonferroni corrected *p* values, adjusting for multiple comparisons.

***p < 0.001, **p < 0.01, *p < 0.1.

^a^ Hb <110 g/L was measured in one donor at first PLT apheresis (106 g/L) and in two at the last apheresis (106 g/L and 109 g/L).

### Course of Hemoglobin and Ferritin

Overall, a decline of median Hb was observed from the first whole blood donation to the switch, with values increasing thereafter (*p* < 0.001 for both comparisons). Ferritin displayed a similar course, with the highest median values being measured at the first whole blood donation and the lowest at the switch (*p* < 0.001) followed by a partial but statistically significant recovery (*p* = 0.001) (Table 1). When focusing on the effect of age, a similar course of Hb and ferritin was observed in women who were 50 years or older at the last PLT donation, while the recovery of both Hb (*p* < 0.01) and ferritin (*p* < 0.1) was not significant between the first and the last PLT donation in the donors aged < 50 years.

Over the course of 30 PLT donations and adjusted for donor age, an overall increasing trend was observed for Hb (*p* = 0.000) (Fig. 1a) that reached statistical significance in each group but was more pronounced for the donors with LowHbFerr (*p* = 0.001). The Hb recovery from the first to the second PLT donation was also more prominent in the LowHbFerr group (+8.4 g/L; 95% CI: 6.1, 10.8, *p* < 0.000), while it had only a borderline significance in both the LowHb (+5.2 g/L; 95% CI: −0.35, 10.8 g/L, *p* < 0.10) and the LowFerr group (−2.7 g/L; 95% CI: −5.4, 0.02, *p* < 0.10) (Fig. 2 a, b). While significant differences in mean Hb during repetitive PLT donations were observed within each group (LowHb vs. LowFerr: −4.9 g/L; 95% CI: −9.0, −0.65; *p* < 0.05 and LowHbFerr vs. LowFerr −6.2 g/L; 95% CI: −9.1, −3.3; *p* < 0.001), the mean Hb increase was not different when comparing groups: 0.26 g/L for LowHb vs. LowFerr (95% CI: −0.11, 0.63; *p* = 0.173) and 0.11 g/L for LowHbFerr vs. LowFerr (95% CI: −0.6, 0.28; *p* = 0.204).

**Fig. 1.**
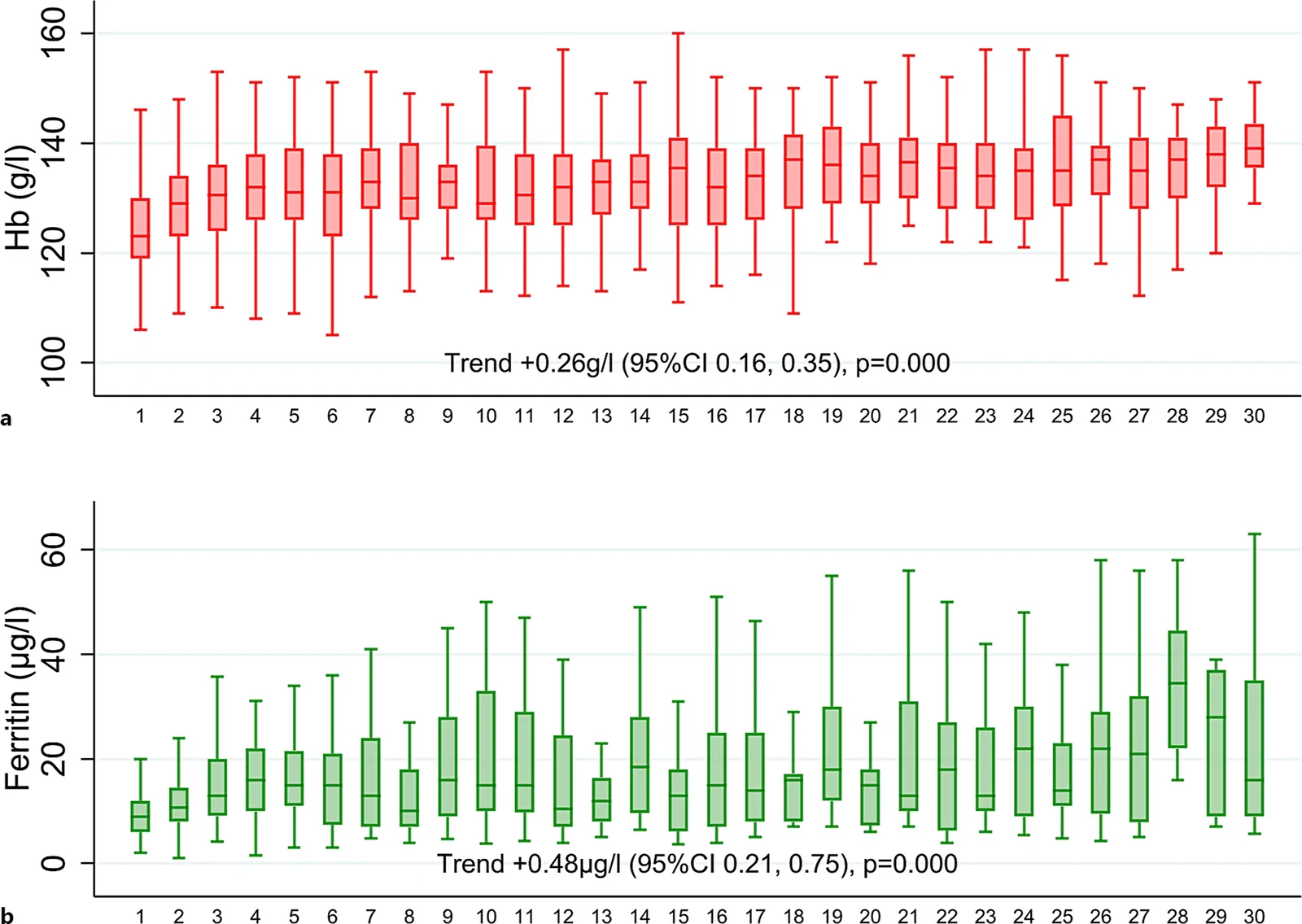
Course of hemoglobin (**a**) and ferritin (**b**) in the whole study cohort over 30 PLT donations after the switch from whole blood donation to PLT apheresis. Trends were estimated using GEE models with robust standard errors, adjusting for donor age.

**Fig. 2.**
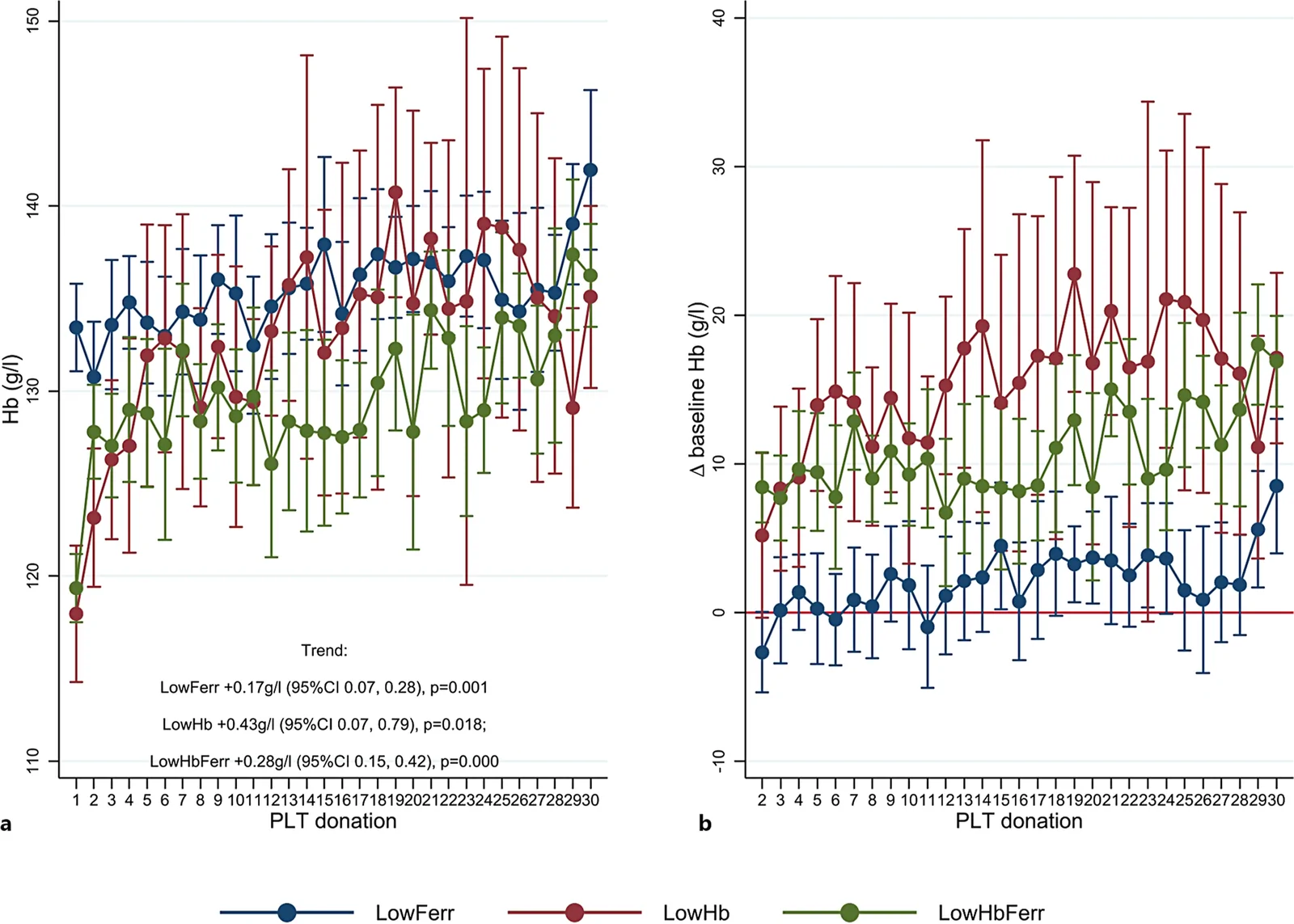
Course of hemoglobin (**a**) and hemoglobin baseline difference (**b**) estimated by reason for donation change over 30 PLT donations after the switch from whole blood donation to PLT apheresis. Estimates are from GEE models with robust standard errors, adjusting for donor age.

When adjusting for age, mean ferritin increased overall (*p* = 0.000) (Fig. 1b). Ferritin increments were greatest in the LowFerr group (*p* = 0.002), reached only a borderline significance in the LowHbFerr group (*p* = 0.073), and were not significant in the LowHb group (*p* = 0.176) (Fig. 3 a, b). Similarly to the Hb changes, differences of mean ferritin levels and mean ferritin increase over repetitive PLT apheresis were not statistically significant in the inter-group comparison. Finally, a significant ferritin drop from the first to the second PLT apheresis was observed only in the LowHb group (−12.3 µg/L; 95% CI: −18.2, −6.4; *p* < 0.001) (Fig. 3 a, b).

**Fig. 3.**
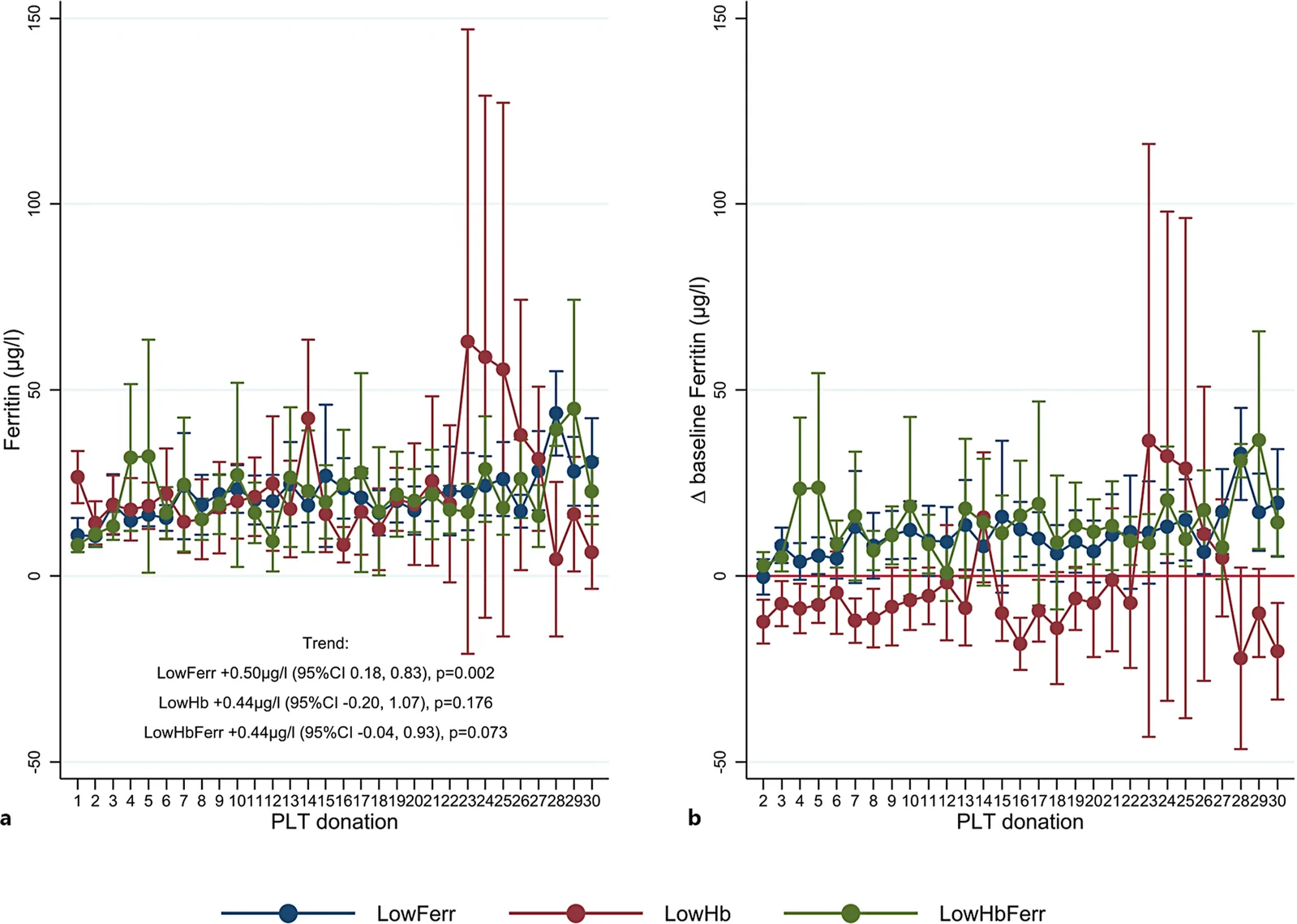
Course of ferritin (**a**) and ferritin baseline difference (**b**) estimated by reason for donation change over 30 PLT donations after the switch from whole blood donation to PLT apheresis. Estimates are from GEE models with robust standard errors, adjusting for donor age.

#### Donation Activity and Retention

Table 2 reports the results of donation activity. In the evaluated period, a total of 2,363 PLT aphereses were performed in the study cohort with the majority being double-dose unit collections, resulting in the procurement of a total of 4,423 PLT units. The median number of PLT donations per donor was 10 (range: 1–155) with a median of 2.3 donations (range: 1–9.2)/donor/year. The period of active donation per donor was over 7 years in median.

**Table 2.** Blood donation activity and donor retention of the study cohort (*N* = 85)

Duration of blood donation activity	
Overall years, *n*	1,024
Years of active blood donation per donor, median (range), *n*	7.2 (0.8–29.5)
Plateletpheresis and PLT products	
Overall PLT donations, *n*	2,363
PLT donations per donor, median (range), *n*	10 (1–155)
PLT donations per year/donor, median (range)	2.3 (1–9.2)
Single-dose PLT donations, *n*	303 (13%)
Double-dose PLT donations, *n*	2,060 (87%)
Total PLT units, *n*	4,423
Donor retention at last follow-up	
Active blood donors, *n*	61 (72%)
Ongoing donation by PLT donations, *n*	56 (66%)
Change back to whole blood donation, *n*	5 (6%)^a^
Permanent deferral, *n*	8 (9%)^b^
Lost to blood donation, *n*	9 (11%)

^a^ Donor wish, adverse event of apheresis, difficult venous access, platelet count < 150 G/L.

^b^ Donor wish (*N* = 4), heart disease (*N* = 2), anemia (*N* = 1), severe adverse event of donation (*N* = 1).

As of December 2023, 61 (72%) of the women of the study cohort were still donating. The majority continued donating PLT by apheresis, while a few switched back to whole blood donation. Eight women were permanently deferred because of donor decision (No. 4), arrhythmia (No. 1), persistent anemia (No. 1), cardiac surgery (No.1), and severe adverse event of donation (No. 1). For nine donors, the reason for abandoning blood donation was not known.

Over the first 24 months of PLT donations, five donors stopped donating PLT for any of the reasons listed above. At the end of a 24-month period, 94% (95% CI: 86.4%, 97.5%) of the cohort were actively donating PLT (Fig. 4a). The percentage of active PLT donors was 94% (95% CI: 77.3, 98.4), 91% (95% CI: 50.1, 98.7), and 95% (95% CI: 82.3, 98.8) in the LowFerr, LowHb, and LowHbFerr group, respectively (Fig. 4b). The log-rank test as well as the Wilcoxon test did not indicate significant differences between groups.

**Fig. 4.**
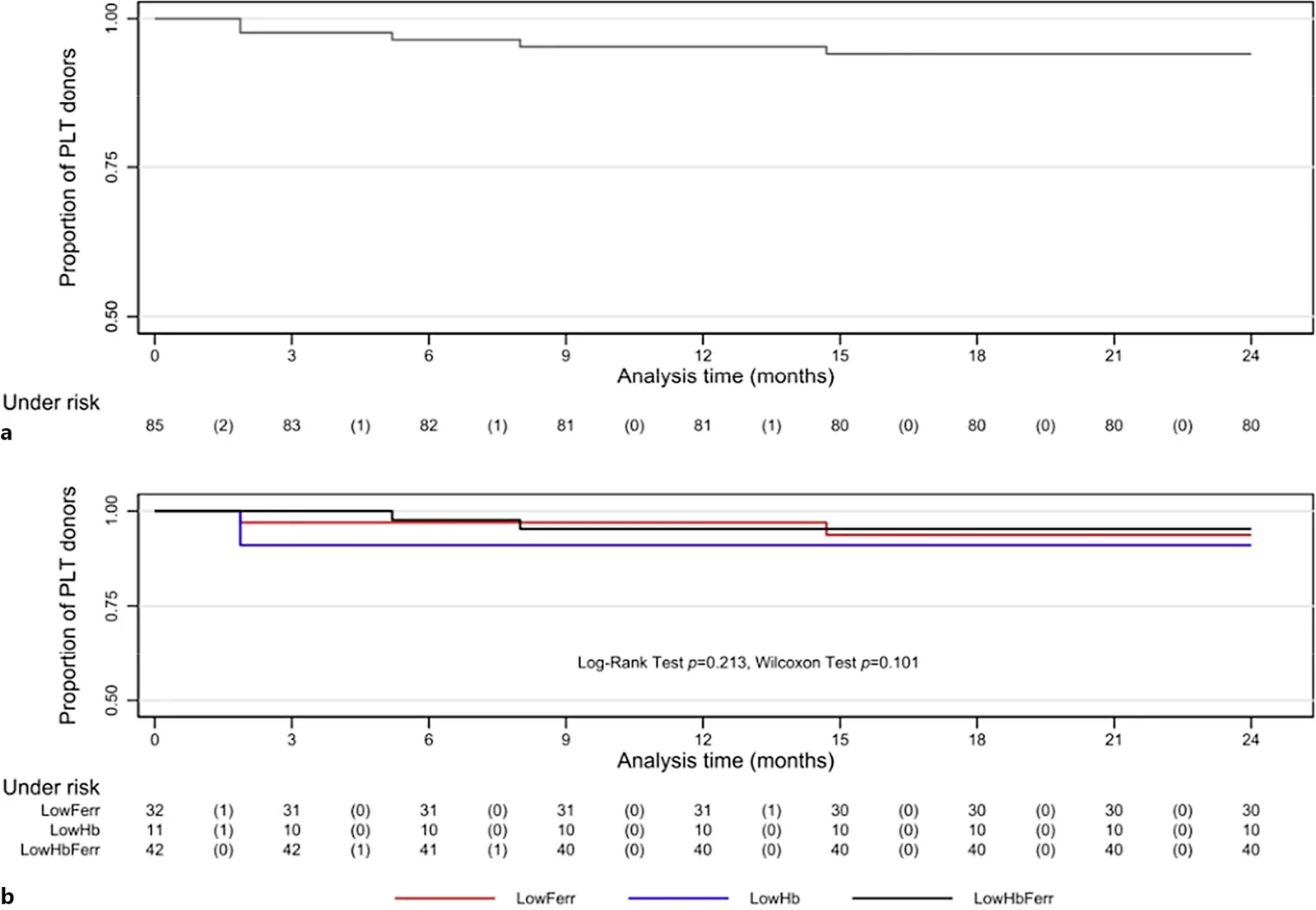
Kaplan-Meier estimator of active donors at 24 months in the whole cohort (**a**) and by reason for donation change (**b**). The proportion of active PLT donors at the end of a 24-month period was 94% (95% CI: 86.4%, 97.5%).

## Discussion

We evaluated retrospectively the course of Hb and ferritin and the retainment to active blood donation in a cohort of 85 women who were ineligible for whole blood donation either because of low Hb or because of an elevated risk of ID and anemia and who were accepted for PLT apheresis. Over many years of observation and with a median frequency of 2.3 PLT apheresis/year/donor, an overall increase of Hb and ferritin was observed. At the end of a 24-months period of PLT donation, the majority of the women in the cohort were still registered as active donors. Our policy proved to be protective for a donor population particularly prone to develop ID and anemia and to be effective in ensuring high donation adherence at the same time.

This longitudinal evaluation extends and confirms our previous data [23] and corroborates the results of the few published studies on a similar strategy [19, 20]. We believe that some results of our study are of particular interest.

First, as a novel aspect, our analysis provides a description of adjusted ferritin changes over a long period of PLT donation (seven years per donor in median). We demonstrate that low frequency PLT apheresis is not only safe regarding iron loss but also allows a partial recovery of already depleted iron stores in donors and that switching to PLT apheresis represents a particular advantage in women with mild or borderline anemia and ID (group LowHbFerr). A similar development can be expected to occur also with regular low-frequency plasma donations [12].

A ferritin cutoff of less than 15 µg/L used in our study and in previous studies as well [24, 25] aligns with the limits recommended by the WHO for defining ID in apparently healthy individuals [5]. However, in the ferritin-based large studies conducted in the Netherlands, the trigger for intervention (prolonging donation intervals and/or offering iron substitution to donors) was set at 30 µg/L [6, 10]. A large NHANES cross-sectional study identified ferritin levels of 25 µg/L as the cutoff associated with indicators of iron-deficient erythropoiesis in non-pregnant women [26], and the same limit for ID was also indicated in an analysis on 286 pre-menopausal female blood donors included in the REDS-RISE data [27]. Therefore, considering these higher ferritin cutoffs, ID was most probably the underlying cause of low Hb also in the 11 women of the LowHb group in our study. Moreover, higher ferritin cutoffs would identify many more whole blood donors, both males and females, having low iron stores, in particular if belonging to the risk groups for ID (e.g., younger individuals, regular donors with Hb close to the cutoff) [13]. Proposing an early switch to PLT or plasma apheresis to these volunteers would likely prevent many deferrals for low Hb. As it was the case for some of the women in our cohort, an alternation of whole blood and apheresis donation, both at low frequency (i.e., 1–2 whole blood donations and 3–4 apheresis donation/year), can be an option depending on specific donor characteristics (e.g., blood group, PLT count, venous peripheral access). In our experience, measuring ferritin at all first donation and monitoring regular whole blood donors proved to be an effective measure for individualized counseling on the type of donation also for men with decreasing ferritin values and for first-time male donors. In fact, this strategy has been established as the principal way of recruiting PLT donors at our center.

The interpretation of the actual protective effect of PLT apheresis in our study is limited by the increasing proportion of post-menopausal women in the cohort over time. We observed that the Hb and ferritin recovery was less pronounced in the women aged 18–50 years, which was an expected result due to the ongoing menstrual loss in younger females. As the median age of the cohort and the number of post-menopausal donors increased, the recovery of Hb and ferritin was greater, mirroring the generally lower risk of ID and anemia in healthy post-menopausal females.

A further significant result is the high retainment (94% as evaluated over any 24-months period) and the strong commitment of the women donating PLT over a long period of time. At the end of the whole observation period, over 70% of the subjects of the cohort were still actively donating, with a median time of PLT donation exceeding seven years per donor. One donor only was permanently deferred because of anemia, while five were able to switch back to whole blood donation.

At our center, the median total number of PLT from apheresis collected between 2019 and 2023 was 2,075 per year, with a sharp increase of requirements from 2022. We calculated that about 215 PLT products were donated yearly by the subjects of the study cohort, corresponding to a proportion of about 10% of the total PLT products per year (data not shown), a nonnegligible contribution to the PLT supply even with a donation frequency of 2–3 apheresis/year. Importantly, 87% of the PLT apheresis in our cohort were double-dose collections. As further implications, the results of our study indicate that these low-frequency PLT donors can be safely asked for additional donations in certain circumstances (e.g., if HLA- or HPA-matched products are required or in case of PLT shortages) and that healthy persons with thalassemia minor can also be eligible for PLT apheresis, a fact of particular relevance for blood establishments in regions with a high prevalence of hemoglobinopathies [28].

Based on these considerations and in the face of the shrinking numbers of active blood donors [29], lowering the Hb acceptance threshold for PLT apheresis represents a feasible and safe way to acquire and retain volunteers who would be otherwise permanently deferred. We believe that our experience can be helpful even to blood centers where ferritin is not regularly measured and can indicate a practical and feasible approach for preserving and possibly enlarging a local blood donor base.

The main limitations of our study are those inherent in its retrospective design. The principal drawbacks are represented by the proportion of missing ferritin values, in particular at baseline and at the time of the change of donation type, the measurements of ferritin based on individual medical judgment not following a standard procedure, and the lack of information on iron substitution in the women of the cohort. We acknowledge that the fact that an indeterminate number of women of the study cohort probably had iron supplementation is a potentially relevant confounder. On the other hand, we describe a real-world experience of a blood donation center where both operational issues and the dependence on the donors’ declarations can hinder a reliable collection and documentation of medical information. Nevertheless, even taking all these limitations into account, our long-standing strategy has proven to be effective in preserving our pool of active volunteers. In our opinion, the strength of our study lies precisely in the consistency of a ferritin-based policy of individualized donor counseling over many years.

In conclusion, we demonstrated that low-frequency regular PLT donations can be safely proposed to otherwise healthy women with low Hb and ferritin, that these measure does not aggravate anemia or ID, and that these donors can be retained in the long term and can contribute significantly to the procurement of PLT products.

## Statement of Ethics

This study protocol was reviewed and approved by the Ethikkommission Nordwest- und Zentralschweiz, Swissethics, (Business Administration System for Ethics Committees, BASEC), Approval No. 2024-01099. Before the inclusion in the study, all participants had given their written consent for the use of data and/or materials related to their blood donation for research purposes (Generalkonsent) as defined by the Swiss Blood Service (V2 of 01.08.2024).

## Conflict of Interest Statement

Andreas Buser was a member of the journal’s Editorial Board at the time of submission. The other authors have no conflicts of interest to declare.

## Funding Sources

This study was not supported by any sponsor or funder.

## Author Contributions

L.I. and V.P. designed the study and collected the data. L.I. wrote the manuscript. T.V. performed the statistical analysis, contributed to the interpretation of the results, and revised the manuscript. A.H., G.S., and A.B. revised the manuscript.

## Data Availability

All data generated or analyzed during this study are included in this article. Further inquiries can be directed to the corresponding author.
